# Organ and tumor dosimetry of [^161^Tb]Tb-PSMA-617 radioligand therapy in patients with mCRPC

**DOI:** 10.1007/s00259-026-08171-z

**Published:** 2026-09-08

**Authors:** Andrea Schaefer-Schuler, Arne Blickle, Sebastian Ganz, Sven Petto, Caroline Burgard, Tilman Speicher, Moritz B. Bastian, Stephan Maus, Mark Bartholomä, Samer Ezziddin, Florian Rosar

**Affiliations:** 1 Department of Nuclear Medicine, Saarland University - Medical Center, Kirrberger Str. 100, Geb. 50, D-66421 Homburg, Germany; 2 Department of Nuclear Medicine, Friedrich-Alexander-Universität Erlangen Nürnberg and Universitätsklinikum Erlangen, Erlangen, Germany

**Keywords:** Tb-161, ^161^Tb, Dosimetry, PSMA, Radioligand therapy, mCRPC, Prostate cancer

## Abstract

**Purpose:**

Terbium-161 (^161^Tb) has emerged as a promising radionuclide for radioligand therapy (RLT) due to its emission of Auger and conversion electrons, which may support therapeutic efficacy through increased local energy deposition. Despite growing research efforts aimed at the clinical potential of ^161^Tb, dosimetric data, particularly for tumor lesions, remain scarce. To address this gap, we assessed tumor and organ dosimetry in mCRPC patients treated with [^161^Tb]Tb-PSMA-617.

**Methods:**

The study involved *n* = 15 patients with metastasized castration-resistant prostate cancer (mCRPC) participating in a registry (NCT04833517) and receiving [^161^Tb]Tb-PSMA-617. All patients received a median of 2 (range: 1–3) cycles with mean administered activity of 6.0 ± 1.3 GBq and mean cumulative activity of 12.3 ± 4.6 GBq [^161^Tb]Tb-PSMA-617. Hybrid dosimetry of organs at risk, including the salivary glands, the kidneys, the liver, and tumor lesions was performed analyzing whole-body planar and SPECT/CT images. Absorbed doses were estimated using OLINDA/EXM for ^161^Tb. Tumor-to-organ ratios (TR) of mean tumor-absorbed doses over relevant organs at risk were calculated.

**Results:**

Mean absorbed dose to kidneys, parotid glands, submandibular glands and liver was 0.70 ± 0.41 Gy/GBq, 0.40 ± 0.20 Gy/GBq, 0.36 ± 0.14 Gy/GBq and 0.13 ± 0.08 Gy/GBq, respectively. Tumor kinetics in 43 lesions demonstrated stable activity over time (mean half-life of all lesions: 44.07 h ± 15.88 h) delivering a mean overall absorbed tumor dose of 5.95 ± 3.60 Gy/GBq, the resulting TR over kidney and parotid gland were 10.55 ± 8.32 and 16.15 ± 11.85, respectively.

**Conclusion:**

[^161^Tb]Tb-PSMA-617 delivers high tumor-absorbed doses with tolerable doses to relevant organs at risk, translating into favorable tumor-to-normal-organ ratios. Dosimetry results confirm ^161^Tb as a promising radionuclide for PSMA-targeted radioligand therapy in mCRPC.

**Supplementary Information:**

The online version contains supplementary material available at https://doi.org/10.1007/s00259-026-08171-z.

## Introduction

As one of the most frequently diagnosed malignancies in men, prostate cancer constitutes a substantial cause of cancer-related mortality worldwide [1]. With disease progression, many patients ultimately develop metastatic castration-resistant prostate cancer (mCRPC), defined by resistance to androgen deprivation therapy (ADT) [2–4]. In the mCRPC setting, prostate-specific membrane antigen (PSMA)-targeted radioligand therapy (RLT) using the beta-emitting isotope lutetium-177 in form of [^177^Lu]Lu-PSMA-617 has demonstrated efficacy, safety, and good tolerability across multiple clinical studies [5–11]. These findings led to regulatory approval by both, the European Medicines Agency (EMA) and the U.S. Food and Drug Administration (FDA) for patients previously treated with at least one line of taxane-based chemotherapy and androgen receptor signaling inhibitors (ARSI). Despite its established role in the treatment of mCRPC, PSMA RLT with ^177^Lu does not achieve responses in all patients. The pursuit of alternative radionuclides therefore represents a rational strategy to enhance antitumor efficacy and address treatment resistance. Alongside alpha emitters like actinium-225, Auger electron emitters, especially terbium-161, have recently gained significant scientific interest as novel candidates for PSMA RLT [12–15]. ^161^Tb appears to be well suited for clinical application as it shares similar chemical properties and physical decay characteristics with the established radionuclide ^177^Lu. The two radionuclides exhibit closely aligned half-lives (^161^Tb: 6.906 days vs. ^177^Lu: 6.647 days), comparable mean β⁻ particle energies (154 keV vs. 133 keV), and γ-emissions of suitable energy to permit post-therapeutic imaging and dosimetric assessment (74.6 keV at 10.2% vs. 208.4 keV at 10.4%). Compared to ^177^Lu, ^161^Tb emits a higher proportion of abundant conversion and low-energy Auger electrons. Their ultra-short tissue range (< 500 nm) is associated with high linear energy transfer (LET; 4–26 keV/µm), promising enhanced local energy deposition [16, 17]. Consequently, ^161^Tb is increasingly and successfully implemented for PSMA RLT in mCRPC in clinical practice and trials, with the first promising clinical results being available [12–15, 18].

Preclinical investigations, underlined by theoretical calculations and simulation studies, have suggested a dosimetric superiority of ^161^Tb compared with ^177^Lu [19–22]. In the first intra-individual head-to-head comparison of [^161^Tb]Tb-PSMA-617 and the standard therapy [^177^Lu]Lu-PSMA-617 conducted in six patients, these findings were corroborated, demonstrating a dosimetric advantage in favor of ^161^Tb [23]. However, dosimetric data for [^161^Tb]Tb-PSMA-617 in large-scale patient cohorts are currently lacking and represent an important, unmet clinical need. The present study aims to address this gap by evaluating dosimetric parameters and absorbed doses for both tumor lesions and organs in a larger cohort of patients treated with [^161^Tb]Tb-PSMA-617.

## Materials and methods

### Patients and ethics

15 patients with mCRPC, enrolled in the ‘*prospective registry to assess outcome and toxicity of targeted radionuclide therapy in patients with mCRPC in clinical routine*’ (REALITYStudy; NCT04833517), were included in this study. Participants received [^161^Tb]Tb-PSMA-617 followed by post-therapeutic dosimetric scintigraphic imaging. All individuals were heavily pretreated including multiple lines of systemic therapy for mCRPC and initial PSMA RLT with established radionuclides (^177^Lu, ^225^Ac). Patient characteristics are summarized in Table 1. Treatment with [^161^Tb]Tb-PSMA-617 was administered on a compassionate-use basis under § 13 (2b) of the German Pharmaceutical Act. Prior to therapy, patients were thoroughly informed about the potential risks of novel radiopharmaceuticals, including radiation exposure and possible side effects, and provided written informed consent. All participants consented to publication of anonymized patient data in accordance with the Declaration of Helsinki. The study was approved by the local institutional review board (Ärztekammer des Saarlandes/Saarbrücken, ethics committee permission number 140/17).

**Table 1:** Baseline patient characteristics, at the time-point of first [^161^Tb]Tb-PSMA-617 administration

Patient characteristics	Value
Age	
Median (range)	74 (65–85)
Age ≥ 65 years, n (%)	15 (100)
PSA, in [ng/mL]	
Median (range)	73 (0.38–2148)
ALP, in [U/L]	
Median (range)	87 (24–449)
Hemoglobin, in [g/dL]	
Median (range)	10 (7–14)
< 13 g/dL, n (%)	13 (86.7)
ECOG performance status, n (%)	
0	4 (26.7)
1	11 (73.3)
≥ 2	0 (0)
Sites of metastases, n (%)	
Local recurrence	3 (20.0)
Bone	12 (80.0)
Lymph node	9 (60.0)
Liver	2 (13.3)
Other	1 (6.7)
Prior therapies, n (%)	
Prostatectomy	10 (66.7)
Radiation	11 (73.3)
ADT	14 (93.3)
NAAD	14 (93.3)
Abiraterone	12 (80.0)
Enzalutamide	14 (93.3)
Bicalutamide	6 (40.0)
Chemotherapy	12 (80.0)
Docetaxel	11 (73.3)
Cabazitaxel	4 (26.7)
Caboplatin	2 (13.3)
^ 223^Ra-dichloride	6 (40.0)
PSMA RLT	15 (100)
[^177^Lu]Lu-PSMA-617 RLT	15 (100)
[^225^Ac]Ac augmented [^177^Lu]Lu-PSMA-617 RLT	4 (26.7)
Prior PSMA RLT cycles, median (range)	
[^177^Lu]Lu-PSMA-617	4 (1–11)
[^225^Ac]Ac-PSMA-617	1 (1–3)

*ADT* androgen deprivation therapy, *ALP*  alkaline phosphatase, *ECOG*  Eastern Cooperative Oncology Group, *NAAD*  novel androgen axis drugs, *PSA*  prostate-specific antigen, *PSMA RLT*  prostate-specific membrane antigen targeted radioligand therapy

Radiolabeling of [^161^Tb]Tb-PSMA-617 and subsequent quality control was performed in analogy to the methodology published previously for [^177^Lu]Lu-PSMA-617 [24]. ^161^Tb was supplied as [^161^Tb]TbCl₃ in 0.05 M HCl (TERTHERA B.V., Breda, The Netherlands) and added to PSMA-617 (ABX GmbH, Radeberg, Germany) dissolved in sodium acetate buffer (1.0 M, pH 4.5), resulting in a specific activity of approximately 42 MBq/nmol. The labeling reaction was carried out at 95 °C for 25 min. Product quality was verified by reversed-phase HPLC. Radiochemical yield and purity consistently exceeded 99%. The administered activities were individually adjusted to patient’s specific characteristics such as tumor progression, distribution and extent of tumor burden, body surface, renal function, or blood cell count, as previously described [25].

### Scintigraphic imaging

For dosimetric assessment, scintigraphy was performed following administration of [^161^Tb]Tb-PSMA-617. Imaging was performed using a hybrid SPECT/CT system (Philips BrightView XCT, Philips Medical Systems, Hamburg, Germany) equipped with low-energy high-resolution (LEHR) parallel-hole collimators for which an extrinsic uniformity correction map was acquired as described previously [23, 26]. The photopeak energy window was set at 74.6 keV with a 20% width, as recommended by MIRD 26 [27]. Planar whole-body scintigraphy was performed on days 1, 2, and 3 after injection (approximately 24 h, 48 h, and ≥ 69 h post-injection). In addition, to apply quantitative 2D/3D hybrid dosimetry, on day 1, a SPECT/CT scan of the head and neck region and on day 2, a SPECT/CT scan covering the abdomen from the liver down to the pelvis was acquired. Whole-body planar imaging was performed at a scan speed of 15 cm/min on days 1 and 2 and 12 cm/min on day 3. Data were acquired in a 256 × 1024 matrix with a pixel size of 4.66 × 4.66 mm^2^. For scatter correction of planar whole-body images, the dual-energy window (DEW) technique was applied, using an additional lower scatter window centered at 69.4 keV with a width of 15%. For attenuation correction, a blank scan and a transmission scan were obtained prior to radiotracer injection using a flat phantom filled with solution of ^161^Tb. SPECT imaging comprised 60 projections over 360°. Acquisition time per projection was 20 s at day 1 for head-and-neck SPECT and 30 s at day 2 for abdominal SPECT. Images were reconstructed on a 128 × 128 matrix with a pixel size of 4.66 × 4.66 mm^2^. Low-dose CT imaging was performed at 120 kV and 10 mA using a 256 × 256 matrix (pixel size: 2.33 × 2.33 mm^2^). CT data were utilized to derive an attenuation map at 100 keV, which was converted to the emission energy of 74.6 keV and applied for attenuation and scatter correction of SPECT images. SPECT reconstruction followed MIRD Pamphlets 23 and 26 recommendations using a 3D OSEM algorithm (3 iterations, 8 subsets) with Butterworth post-filtering (0.5 cutoff, 10th order) and a slice thickness of 4.66 mm [27, 28].

### Organ and tumor dosimetry

Data derived from acquired scintigraphy imaging were used to evaluate dose deposition of [^161^Tb]Tb-PSMA-617 in normal organs and tumor manifestations. Dosimetric calculations were conducted in accordance with the MIRD schema and included the kidneys, liver, salivary glands (parotid and submandibular), as well as targeted tumor lesions in bone, lymph nodes and liver. Dosimetry calculations were carried out by two experienced physicists and one experienced nuclear medicine physician following the previously reported hybrid dosimetry methodology [23, 29]. Analysis was performed using the software program packages QDOSE (ABX-CRO, Dresden, Germany) and OLINDA/EXM version 1.0. All planar whole-body images were quantified on a pixel-wise basis using the conjugate-view method and co-registered employing the rigid registration approach. Background activity was corrected by calculating the activity per pixel within the respective background region of interest (ROI) and subtracting it from the activity in the organ ROI. Manual organ delineation on anterior and posterior images was carried out applying threshold-based segmentation to define the organ boundaries.

For tumor dosimetry, up to 3 target lesions per patient were identified on planar scintigraphic images, with selection restricted to lesions not overlapping with regions from adjacent tissues and exhibiting adequate physiological tracer uptake at all three imaging time points. Furthermore, only lesions that were included within the limited field-of-view of the SPECT acquisition were considered eligible for analysis. Time-activity curves were established through mono-exponential regression of the sequentially measured activities, employing a weighted least squares fit.

Within SPECT/CT datasets, organ- and lesion-specific boundary volumes of interest (VOIs) were manually drawn to fully capture the target structures while avoiding adjacent areas. For each target, the volume and activity within the respective region was subsequently estimated by applying a fuzzy locally adaptive Bayesian (FLAB) algorithm for automatic volume segmentation [30]. The respective camera calibration factor for imaging ^161^Tb was used to convert the arbitrary voxel values into activity. All results were partial-volume corrected using the respective volume-dependent recovery coefficients which were determined by ^161^Tb-based phantom measurements as described in [26]. Within the hybrid 2D/3D dosimetry approach [29], SPECT-derived activity values serve to rescale planar imaging–based time–activity curves. The time-integrated activities were determined as the area under the curve by numeric integration and used to calculate the respective time-integrated activity coefficients [29]. Applying OLINDA/EXM, absorbed doses to tumor lesions and salivary glands were calculated with the spheres model, whereas the reference man was applied for kidneys and liver. Volumes of target lesions were assessed from pre-therapeutic [^68^Ga]Ga-PSMA-11 PET/CT images by drawing VOIs with an isocontour of 20–40% of SUVmax using a dedicated workstation (Syngo.Via, Siemens Healthineers, Erlangen, Germany). Organ masses for the salivary glands were derived from recommendations of ICRP, with estimated weights of 25 g for the parotid gland and 12.5 g for the submandibular gland [31]. Along with absolute absorbed doses, organ-to-tumor dose relationships were analyzed using the tumor-to-organ ratios (TR). The TR was defined as the mean radiation dose absorbed by tumor lesions divided by the mean absorbed dose of a critical organ-at- risk. TR values were obtained for the kidneys and the parotid glands.

### Association between dosimetry and clinical outcome

To evaluate the biochemical response to [^161^Tb]Tb-PSMA-617 RLT, serum PSA levels were measured at baseline (defined as the value obtained at the time of the first administration of [^161^Tb]Tb-PSMA-617) and compared with the lowest PSA value recorded during or after PSMA RLT. Biochemical response was defined according to Prostate Cancer Clinical Trials Working Group 3 criteria [32] with partial response (PR) defined as a decrease ≥ 50% and biochemically stable disease (SD) as an intermediate change (< 50% decrease to < 25% increase). Biochemical progressive disease (PD) was defined as a relative PSA increase of ≥ 25%. The relationship between biochemical response and dosimetry results was assessed by analyzing the correlation between %PSA change and mean tumor lesion absorbed dose.

### Statistical analysis

All continuous data reported is expressed as mean, standard deviation, median and range. Normality testing indicated that none of the recorded data sets were normally distributed. The difference between absorbed dose after the first and the following cycles was evaluated using a Wilcoxon matched-pairs signed ranks test. A nonparametric unpaired Mann-Whitney test was performed to test for difference of lesion absorbed dose depending on metastatic site. Differences in mean lesion absorbed dose per patient depending on PSA response following PCWG 3 criteria were assessed by a nonparametric unpaired Kruskal-Wallis test (one-way ANOVA analysis). Spearman’s rank correlation coefficient was calculated to evaluate the correlation between these metrics. All statistical analyses were carried out using GraphPad Prism (GraphPad Software; Version 11.0.0, San Diego, USA).

## Results

**Fig. 1:**
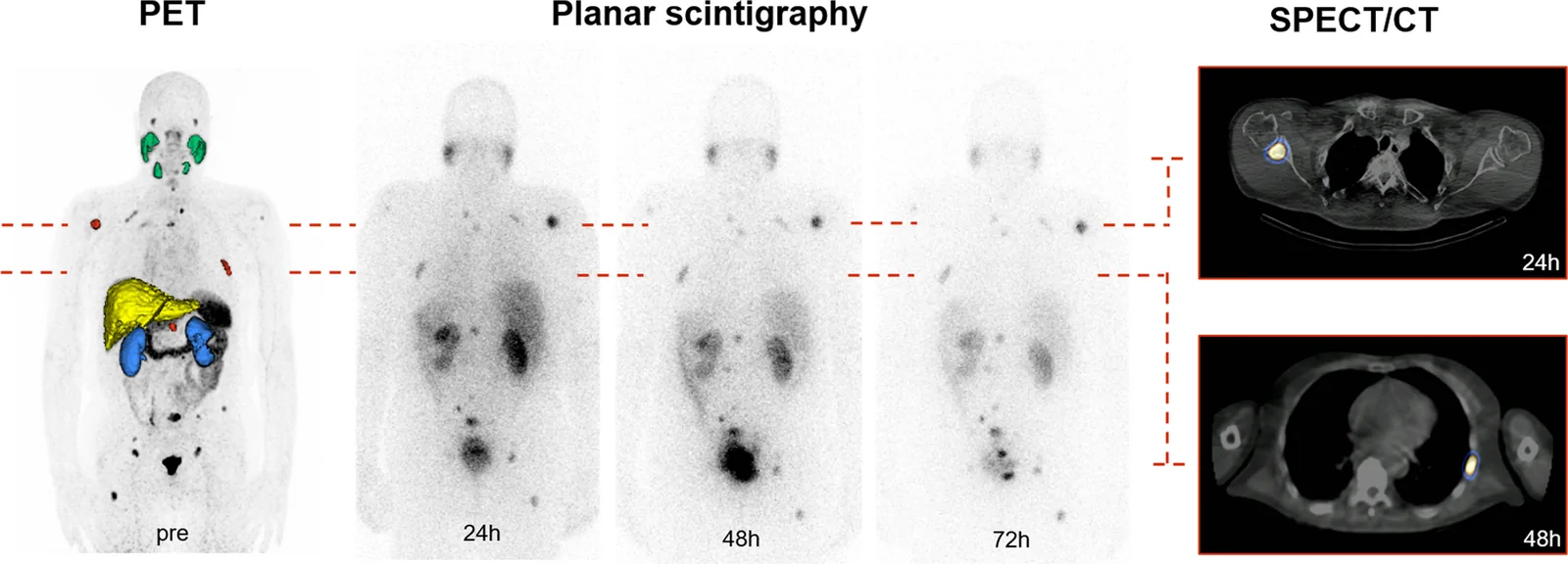
Pretherapeutic [^68^Ga]Ga-PSMA-11 PET maximum-intensity-projection (anterior view) and post-therapeutic scintigraphic [^161^Tb]Tb-PSMA-617 imaging from one representative patient including planar whole-body images approximately acquired 24 h, 48 h, and 72 h p.i. (posterior views) and SPECT/CT 24 h and 48 h p.i. Analyzed organs: salivary gland (green), liver (yellow), kidneys (blue) demonstrating decreasing uptake over time. Target lesions of metastatic disease (red) are showing long-term retention

Across the 15 mCRPC patients included in this study a total of 30 cycles of [^161^Tb]Tb-PSMA-617 RLT were analyzed regarding dosimetry of normal organs and metastatic lesions. The mean administered activity per cycle was 6.0 ± 1.3 GBq (range: 4.3–7.8 GBq), with a median of 2 cycles per patient (range: 1–3 cycles). Alongside dosimetry of organs, including the salivary glands, the kidneys, and the liver, dosimetric analysis was performed for overall 43 metastases, with a median of 3 (range: 2–4) target lesions per patient. Planar whole-body images were acquired 21 ± 3 h, 46 ± 2 h and 84 ± 15 h post injection followed by SPECT/CT imaging 22 ± 3 h and 47 ± 2 h p.i. Figure 1 demonstrates representative biodistribution pattern of radioactively labeled PSMA ligands presenting post-therapeutic scintigraphic planar whole-body images ([^161^Tb]Tb-PSMA-617) together with pre-therapeutic PSMA PET ([^68^Ga]Ga-PSMA-11). The analyzed organs and target lesions are highlighted by color to facilitate identification, illustrating the prolonged retention of [^161^Tb]Tb-PSMA-617 in metastases and the progressive decline in uptake in normal organs over time.

### Normal organ dosimetry

**Fig. 2:**
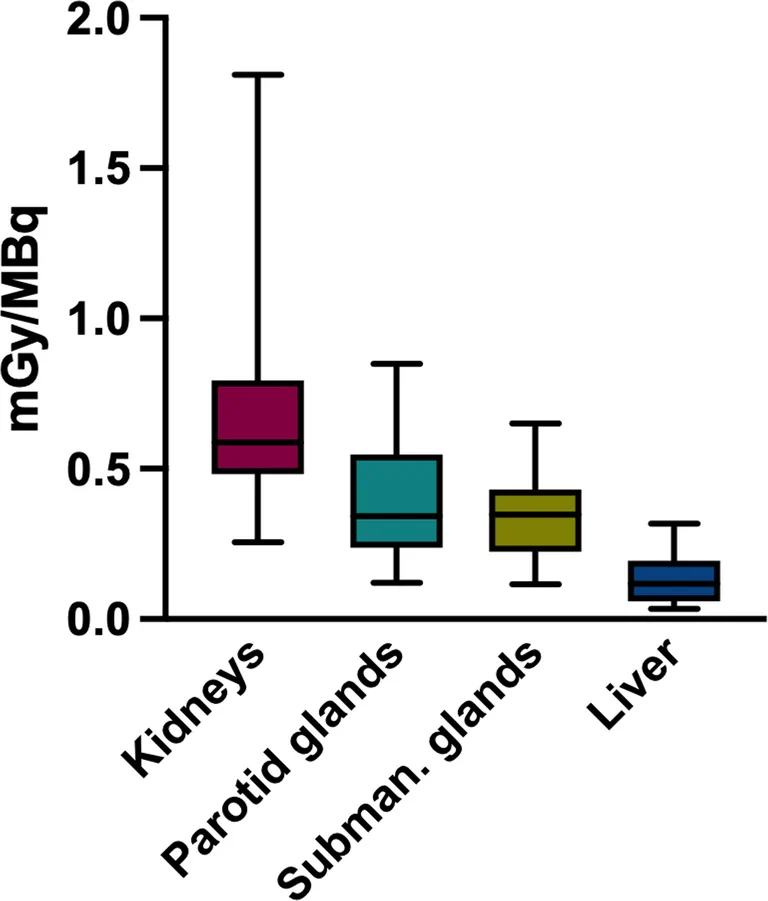
Absorbed dose estimates for the kidneys (median: 0.59 Gy/GBq; range: 0.26–1.81 Gy/GBq), parotid glands (median: 0.34 Gy/GBq, range: 0.12–0.85 Gy/GBq), submandibular glands (median: 0.37 Gy/GBq, range: 0.15–0.65 Gy/GBq), and liver (median: 0.12 Gy/GBq, range: 0.03–0.32 Gy/GBq), calculated over the entire patient-cohort

**Fig. 3:**
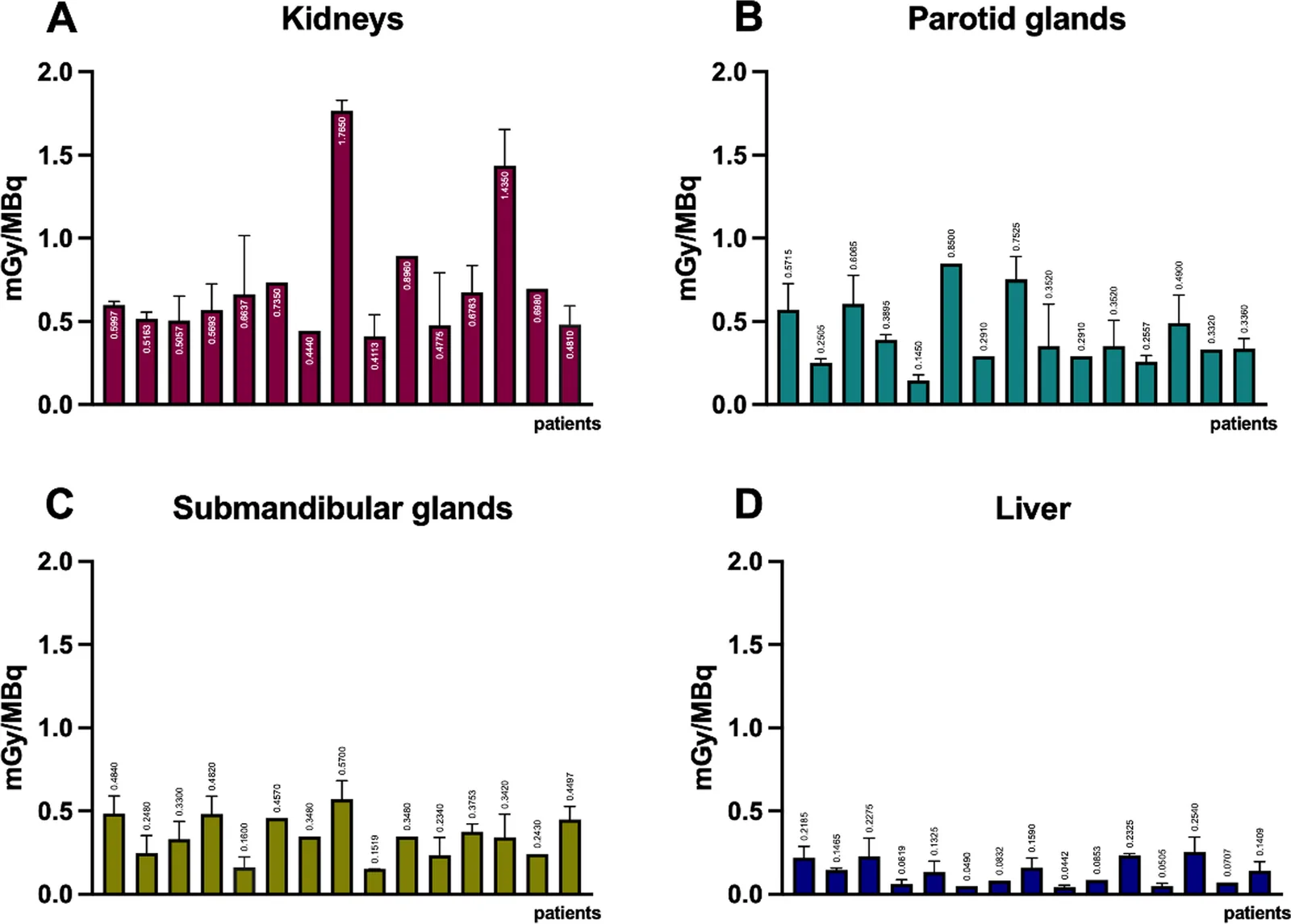
Mean absorbed dose for each patient, shown for evaluated normal organs: **(A)** kidneys (mean: 0.70 ± 0.41 Gy/GBq, **(B)** parotid glands (mean: 0.40 ± 0.20 Gy/GBq), **(C)** submandibular glands (mean: 0.36 ± 0.14 Gy/GBq), and **(D)** liver (mean: 0.13 ± 0.08 Gy/GBq)

Absorbed dose estimates across all administered [^161^Tb]Tb-PSMA-617 cycles as derived from hybrid 2D/3D dosimetry showed the highest mean absorbed dose in the kidneys (0.70 ± 0.41 Gy/GBq, median 0.59 Gy/GBq), followed by the salivary glands (0.40 ± 0.20 Gy/GBq, median 0.34 Gy/GBq for parotid glands and 0.36 ± 0.14 Gy/GBq, median 0.37 Gy/GBq for submandibular glands, respectively). The lowest values were found for the liver (0.13 ± 0.08 Gy/GBq, median 0.12 Gy/GBq) Fig. 2. Interindividual variability was observed across patients, with absorbed dose ranges of 0.26–1.81 Gy/GBq for the kidneys, 0.12–0.85 Gy/GBq for parotid glands, 0.15–0.65 Gy/GBq for the submandibular glands, and 0.03–0.32 Gy/GBq for the liver (Fig. 3 A-D). The respective monoexponential curve-fitting parameters, the time-integrated activity coefficients (TIACs), and the absorbed dose estimates of the normal organs are presented in Table 2. As shown in Figure S1A (supplemental material), the estimated absorbed doses to the investigated normal organs did not differ significantly between the first and second cycle of [^161^Tb]Tb-PSMA-617 RLT (all *p* > 0.187).

**Table 2:** Monoexponential curve-fitting parameters, time-integrated activity coefficients (TIAC) and mean absorbed dose estimates for [^161^Tb]Tb-PSMA-617 in selected organs. Results are presented as mean value ± standard deviation (SD)

Organ	%IA %injected A_0_	^161^Tb-PSMA 617 lambda (h^− 1^)	TIAC (h)	Absorbed dose (Gy/GBq)
Kidneys	2.09 ± 1.32	0.022 ± 0.007	1.74 ± 0.64	0.70 ± 0.13
Liver	3.27 ± 2.43	0.024 ± 0.009	1.41 ± 0.95	0.13 ± 0.08
Parotid gland	0.57 ± 0.29	0.028 ± 0.007	0.16 ± 0.09	0.40 ± 0.20
Submand. gland	0.45 ± 0.21	0.027 ± 0.009	0.07 ± 0.04	0.36 ± 0.14

### Dosimetry of tumor lesions

**Fig. 4:**
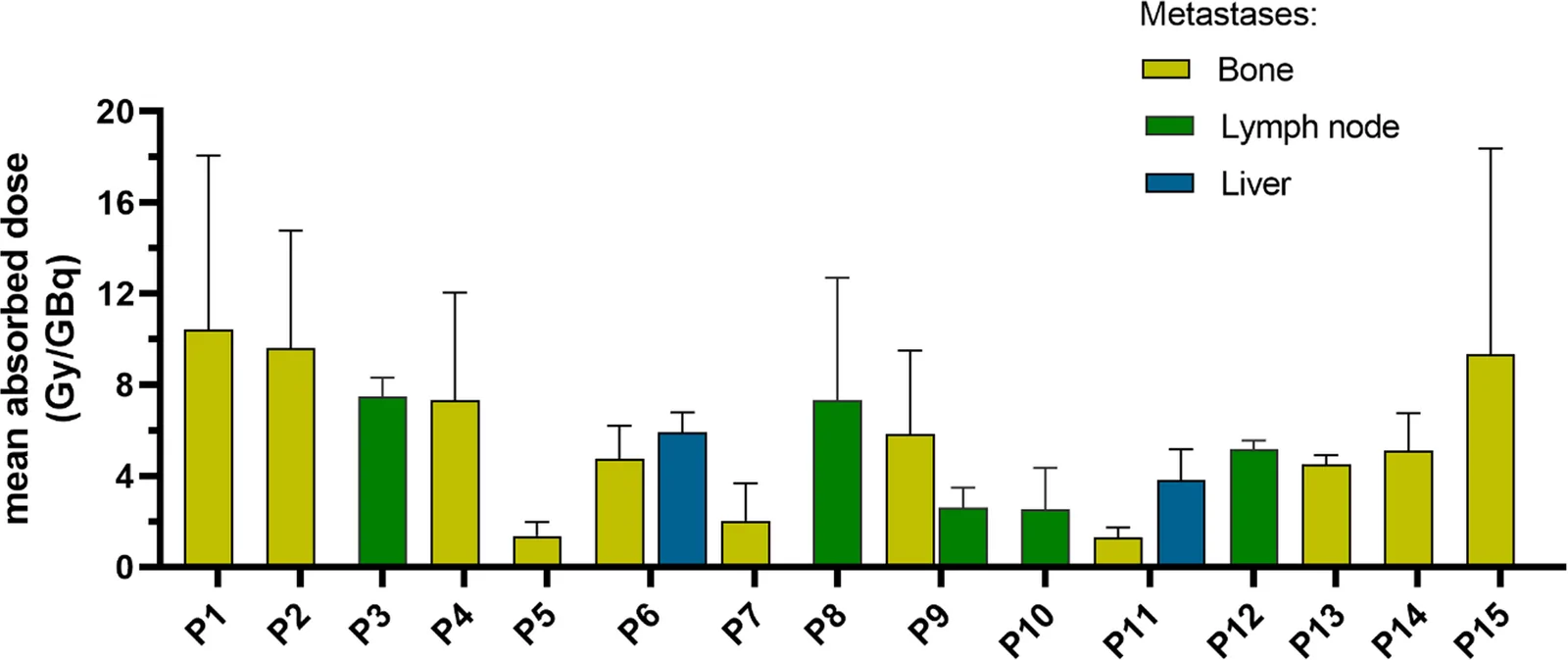
Mean absorbed dose per patient in bone metastases, lymph node metastases and liver metastases determined after application of [^161^Tb]Tb-PSMA 617

Within the cohort of 43 analyzed target tumor lesions, bone metastases predominated, representing 30/43 lesions, involving 11/15 patients. 9 lymph node metastases in 5/15 patients were evaluated and 4 liver metastases in 2/15 patients. The dosimetric analysis of tumor lesions demonstrated substantial intra- and interpatient variability, with estimated absorbed doses ranging from 0.68 to 32.34 Gy/GBq (Fig. 4) and a mean overall absorbed dose in analyzed metastases of 5.95 ± 3.60 Gy/GBq (median: 5.69 Gy/GBq). Additionally, Figure S1B (supplemental material) indicates that differences in tumor lesion absorbed dose estimates between treatment cycles were statistically non-significant (*p* = 0.102).

**Fig. 5:**
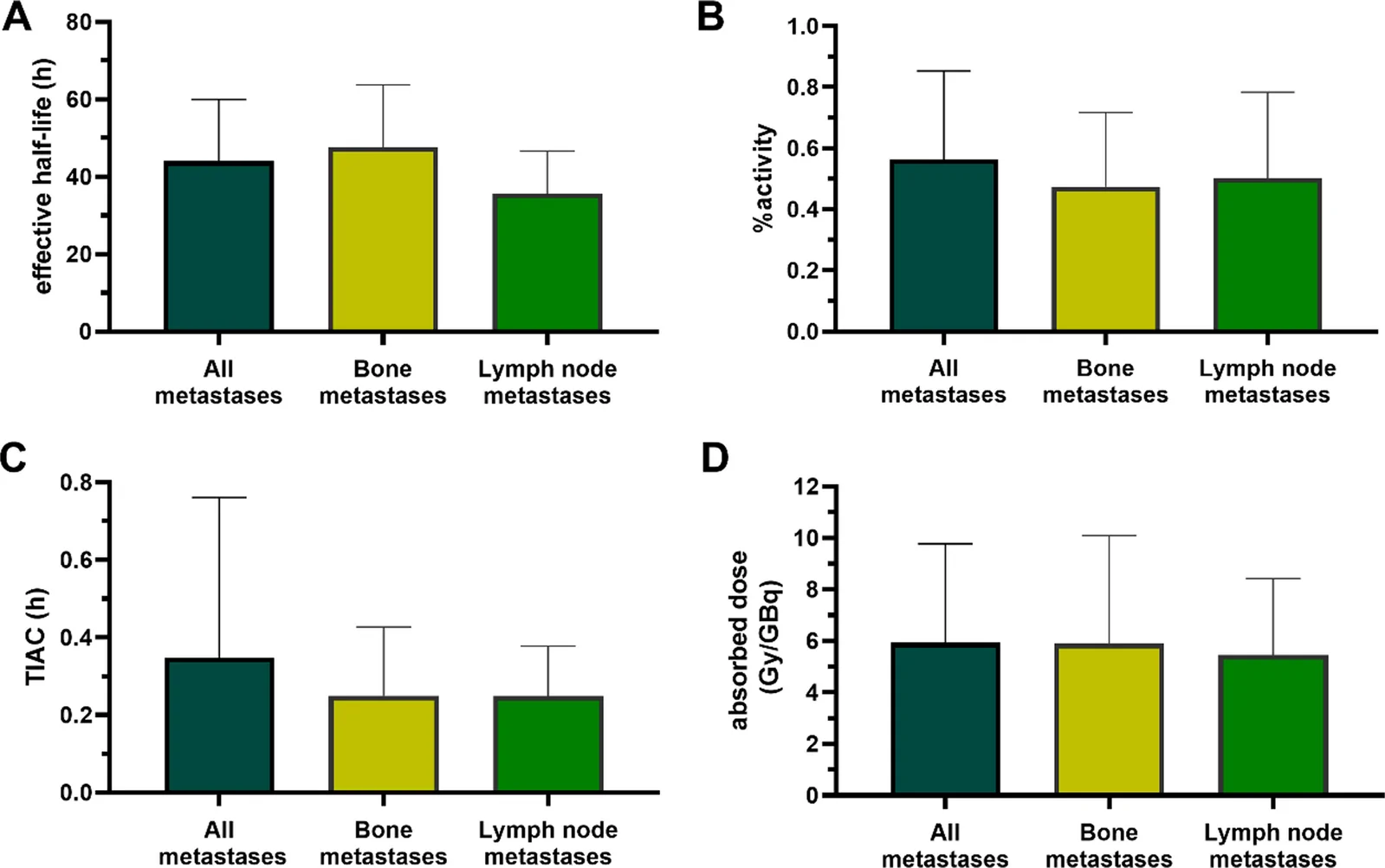
Results of tumor dosimetry. **(A)** Mean effective half-life, **(B)** mean %injected activity, **(C)** mean time-integrated activity coefficients (TIAC) and **(D)** mean absorbed dose estimates for [^161^Tb]Tb-PSMA-617. Results for all tumor lesions evaluated in the patient cohort are presented in comparison to bone metastases and lymph node metastases

Since most investigated lesions involved bone or lymph nodes, these two sites were considered separately in the analysis. Monoexponential fitting of TIAC curves showed mean effective half-life in all tumor lesions of 44.07 h ± 15.88 h with significantly higher effective half-life in bone metastases (T_eff_: 47.63 ± 16.10 h) compared to lymph node metastases (T_eff_: 35.61 ± 11.00 h; *p* = 0.03) (Fig. 5A**)**. The mean %injected activity was comparable between both groups with slightly higher values observed in lymph nodes (bone metastases: 0.47 ± 0.24%; lymph node metastases: 0.50 ± 0.28%; *p* = 0.963) (Fig. 5B). In addition, the two groups showed also similar mean TIACs without a statistically significant difference (bone metastases: 0.25 ± 0.18 h; lymph node metastases: 0.25 ± 0.13 h; *p* = 0.796) (Fig. 5C). Finally, only small, statistically non-significant differences were observed comparing the estimated absorbed doses for bone and lymph node metastases (bone: 5.89 ± 4.20 Gy/GBq, 95%-CI [3.98 7.81] Gy/GBq; lymph node: 5.46 ± 2.97 Gy/GBq, 95%-CI: [3.57 7.34] Gy/GBq; *p* = 0.920; Fig. 5D), while hepatic lesions exhibited lower doses (4.53 ± 1.78 Gy/GBq), 95%-CI [0.18 8.95]. However, this difference also did not reach statistical significance.

**Fig. 6:**
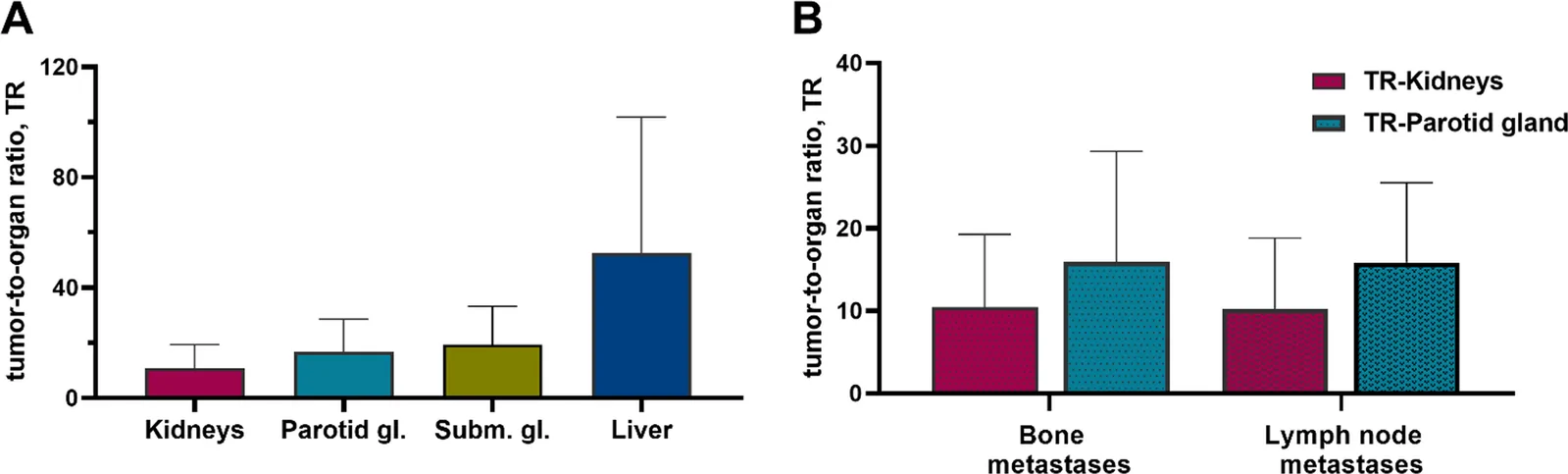
**(A)**: Tumor-to-organ ratio (TR) over kidneys (mean: 10.55 ± 8.32), liver (51.81 ± 47.60), parotid gland (16.15 ± 11.85) and submandibular gland (19.03 ± 13.88) determined for [^161^Tb]Tb-PSMA-617. (**B**) TR of bone metastases and lymph node metastases over the critical organs, the kidneys and the parotid gland

Tumor-to-organ ratios (TR) were derived from mean absorbed tumor doses divided by those of the kidneys, salivary glands, and liver, demonstrating high TR values for all evaluated normal organs. Mean TRs for the kidneys and parotid glands were 10.55 ± 8.32 and 16.15 ± 11.85, respectively. Bone lesions demonstrated slightly higher ratios (TR-kidney: 10.41 ± 8.87; TR-parotid gland: 15.99 ± 13.34) compared with the lower ratios of lymph node metastases (TR-kidneys: 10.25 ± 8.56; TR-parotid gland: 15.87 ± 9.67) and hepatic metastases (TR-kidney: 8.43 ± 1.34; TR-parotid gland: 9.50 ± 2.01). Figure 6A summarizes the mean TRs for all evaluated normal organs, while Fig. 6B separately demonstrates the TRs of bone metastases and lymph node metastases to the organs at risk, the kidneys and the parotid gland.

### Association between dosimetry and clinical outcome

**Fig. 7:**
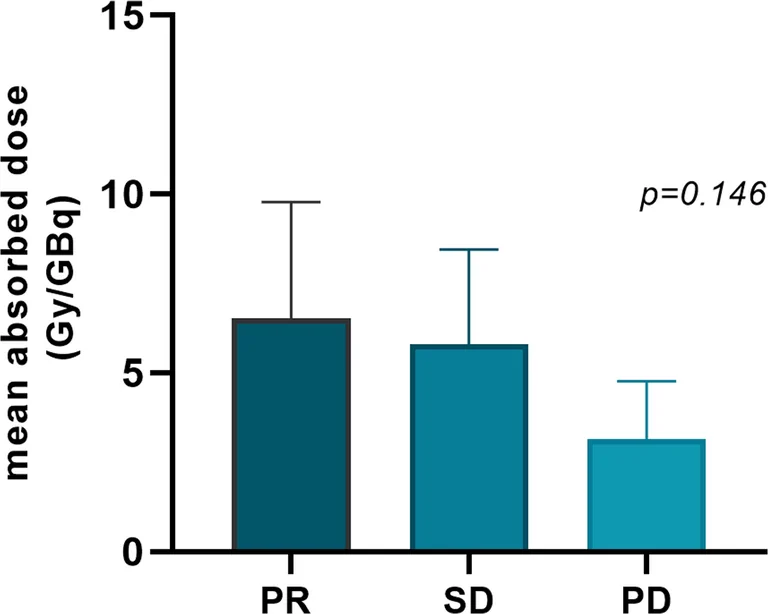
Mean absorbed dose to tumor lesions after one cycle of [^161^Tb]Tb-PSMA-617 for patients showing different PSA response. Partial remission (PR): PSA decrease ≥ 50% (mean: 6.52 ± 3.27 Gy/GBq, 95%-CI [3.27 9.54] Gy/GBq), stable disease (SD): PSA decrease < 50% or increase < 25% (mean: 5.81 ± 2.94 Gy/GBq, 95%-CI [3.03 8.58] Gy/GBq) and progressive disease (PD): PSA increase ≥ 25% (mean: 3.15 ± 1.62 Gy/GBq, 95%-CI [0.56 5.73]). One-way ANOVA revealed *p* = 0.146

The treatment response was assessed biochemically by changes in serum PSA from baseline (the time of the first administration of [^161^Tb]Tb-PSMA-617) during follow-up. The best PSA response following initiation of [^161^Tb]Tb-PSMA-617 RLT was used for the subsequent analysis. Respective results for the three patient groups summarized according to PCWG3 criteria are presented in supplementary material Table S1. Figure 7 presents a comparison of the mean tumor lesion absorbed dose per patient across the different treatment response groups. Although the differences in mean absorbed dose did not reach statistical significance (*p* = 0.146), a trend toward differences among the response groups was observed. Patients with progressive disease tended to have lower tumor absorbed doses (mean: 3.15 ± 1.62 Gy/GBq; 95%-CI [0.56 5.73] Gy/GBq) than patients with stable disease (mean: 5.81 ± 2.64 Gy/GBq; 95%-CI [3.03 8.58] Gy/GBq) or partial remission (mean: 6.52 ± 3.27 Gy/GBq; 95%-CI [3.27 9.54] Gy/GBq). Correlation analysis revealed moderate correlation (*r* = 0.457, *p* = 0.09).

## Discussion

^161^Tb-based PSMA RLT is increasingly recognized as a promising treatment strategy for patients with mCRPC. The present analysis aimed to provide comprehensive real-world dosimetric data for [^161^Tb]Tb-PSMA-617 and, to our knowledge, represents the largest dosimetry study of ^161^Tb-labeled PSMA RLT to date. The analysis demonstrates favorable dosimetry results for both normal organs and tumor lesions, ultimately yielding promising tumor-to-organ dose ratios and thereby underscoring its considerable therapeutic potential.

Normal organ absorbed dose estimates derived from hybrid 2D/3D dosimetry across *n* = 30 treatment cycles of [^161^Tb]Tb-PSMA-617 in 15 patients demonstrated the highest mean absorbed dose in the kidneys (0.70 ± 0.41 Gy/GBq), followed by the salivary glands (parotid glands: 0.40 ± 0.20 Gy/GBq; submandibular glands: 0.35 ± 0.14 Gy/GBq) and the liver (0.13 ± 0.08 Gy/GBq). These findings are consistent with those of our pilot study, which included a smaller cohort of six patients undergoing a single cycle of [^161^Tb]Tb-PSMA-617 [23, 33]. Recently, results from the first Phase I/II VIOLET study evaluating [^161^Tb]Tb-PSMA-I&T have been reported, including preliminary normal organ dosimetry data from nine patients following the first treatment cycle [34]. In comparison with our findings, the authors reported slightly lower absorbed doses for the kidneys (mean: 0.36 ± 0.11 Gy/GBq). Similarly, lower absorbed doses were observed for the submandibular and parotid glands (mean: 0.15 ± 0.07 Gy/GBq and 0.16 ± 0.08 Gy/GBq, respectively), as well as for the liver (mean: 0.06 ± 0.03 Gy/GBq) [14, 35]. These discrepancies compared with our findings may be attributable to differences in the specific PSMA ligand employed and the heterogeneity of the respective patient cohorts investigated. In addition, differences in dosimetric methodologies used to estimate absorbed doses should also be considered, as this may further contribute to interstudy variability [27, 29]. Furthermore, substantial interpatient variability in absorbed dose was observed in the current study on treatment with [^161^Tb]Tb-PSMA-617, with kidney absorbed dose ranging from 0.26 Gy/GBq to 1.81 Gy/GBq. Two patients exhibited particularly high kidney doses (1.59 Gy/GBq and 1.72 Gy/GBq). Both had pre-existing renal risk factors at baseline (both patients were graded CTCAE°3 at baseline), however, no deterioration in renal toxicity was observed over time for these patients.

The comparison of absorbed doses in our cohort for [^161^Tb]Tb-PSMA-617 with well-established data for [^177^Lu]Lu-PSMA-617 indicates that the absorbed doses to normal organs fall within the wide range of values typically reported for this radioligand. For example, renal absorbed doses published for [^177^Lu]Lu-PSMA-617 range from 0.39 ± 0.15 Gy/GBq to 0.99 ± 0.31 Gy/GBq) [36–38]. However, whereas low physiological uptake in critical organs is desirable from a safety perspective, deposition of high radiation doses in tumor lesions is of prominent relevance for optimizing therapy treatment. With regard to tumor dosimetry, the mean absorbed dose observed across 43 metastases (5.95 ± 3.60 Gy/GBq) is consistent with our preliminary published findings of a mean absorbed dose of 6.10 ± 6.59 Gy/GBq in 17 metastases [23]. These results are further supported by site-specific analyses of metastatic involvement. Estimated absorbed doses were 5.89 ± 4.20 Gy/GBq for bone metastases and 5.46 ± 2.97 Gy/GBq for lymph node metastases. For comparison, a systematic review and meta-analysis of [^177^Lu]Lu-PSMA dosimetry (including different PSMA ligands) encompassing 595 patients reported mean absorbed doses of 3.57 Gy/GBq (4.10 Gy/GBq) for bone lesions and 4.19 Gy/GBq (2.94 Gy/GBq) for soft tissue lesions following administration of [^177^Lu]Lu-PSMA-617 (or [^177^Lu]Lu-PSMA-I&T respectively) [38]. Furthermore, in the current study, a substantial variability in lesion absorbed dose (range: 0.6–32.3 Gy/GBq) was observed, resulting in a wide range of mean tumor absorbed doses (1.3–10.4 Gy/GBq) for the individual patient. Notably, this pronounced interpatient variability in tumor dosimetry has also been reported in several previous studies, investigating various ^177^Lu-labeled PSMA ligands. For example, for [^177^Lu]Lu-PSMA-617, Violet et al. [37] reported absorbed dose ranges of 0.41–10.71 Gy/GBq for bone metastases and 0.52–16.23 Gy/GBq for lymph node metastases. In contrast, for [^177^Lu]Lu-PSMA-I&T, Feuerecker et al. [39] described a range of 0.38–9.80 Gy/GBq across both metastatic sites. These findings indicate that mean absorbed dose values may vary considerably between patients, likely reflecting the heterogeneous nature of the study populations. In addition, variability in tumor absorbed dose may also be explained by differences in prior therapeutic interventions specifically prior PSMA RLT. Patients of the cohort were heavily pretreated including PSMA RLT with multiple cycles of ^177^Lu as well as ^225^Ac labeled ligands (compare Table 1). However, in our cohort, the mean radiation doses delivered to metastatic sites by [^161^Tb]Tb-PSMA-617 were higher than those reported for RLT with various ^177^Lu-labeled PSMA-ligands.

Our data demonstrate that the absorbed tumor doses translate into favorable TR of 10.55 ± 8.32 and 16.15 ± 11.85 for the organs at risk, namely the kidneys and the parotid glands. In comparison, published data for ^177^Lu-based PSMA RLT have reported lower kidney therapeutic indices ranging from 3.6 to 5.3, depending on the radioligand used [23, 39]. Overall, the findings confirm that ^161^Tb is a therapeutic radionuclide for PSMA RLT that enables high energy deposition within tumor lesions relative to organs at risk, thereby providing favorable tumor-to-organ ratios. This finding may be attributable to the emission of a substantial number of conversion and low-energy Auger electrons that deposit energy over a much shorter distance compared with beta radiation [16]. Monte Carlo simulations and microdosimetric calculations have shown that these electrons enable highly localized energy deposition and are therefore considered particularly advantageous for the irradiation of small tumor lesions and micro-metastasis [17, 22]. The promising dosimetric properties of ^161^Tb are supported by a growing body of preclinical evidence. In vitro experiments and murine tumor models could demonstrate an enhanced antitumor efficacy of ^161^Tb-based RLT relative to ^177^Lu [20, 21]. Despite these encouraging results, comprehensive microdosimetric measurements are still lacking and clinical experience with ^161^Tb remains limited, with only a small number of published studies predominantly descriptive in nature [14, 15, 40]. Nevertheless, consistent with the favorable dosimetric profile, promising clinical efficacy was observed even in this heavily pretreated patient population, with a substantial proportion of patients achieving partial remission or stable disease. These results are in line with our previously published findings from the initial clinical experience indicating promising outcome following [^161^Tb]Tb-PSMA-617 RLT in a cohort which included the patient cohort analyzed in the present dosimetry analysis [15, 40]. Consistent with these observations, patients achieving partial response or stable disease exhibited a trend toward higher tumor absorbed doses than those with progressive disease. Although this association did not reach statistical significance, most likely due to the limited sample size and the heterogeneous, heavily pretreated study population, it supports the concept of a dose–response relationship in PSMA-targeted RLT [41, 42]. Concerning toxicity, our initial clinical experience could also indicate a favorable safety profile following [^161^Tb]Tb-PSMA-617 RLT in the respective cohort [15]. Correspondingly, no severe adverse events or treatment-related therapy discontinuations were observed in the present cohort.

Preliminary results from the phase I/II VIOLET trial further support the therapeutic potential of ^161^Tb–based PSMA-targeted radioligand therapy, demonstrating that [^161^Tb]Tb-PSMA-I&T is safe, well tolerated, and associated with promising antitumor activity [14]. While direct comparison between our dosimetric findings and the clinical outcomes reported by Buteau et al. should be interpreted with caution, given the heterogeneity of the respective patient cohorts, the high absorbed doses delivered to tumor lesions by [^161^Tb]Tb-PSMA-617 in our study further support its potential therapeutic efficacy in patients with advanced mCRPC [14]. In this context, individualized dosimetry may play an important role in the future by supporting patient-specific treatment planning and adaption, especially for RLT with new radiopharmaceuticals, such as [^161^Tb]Tb-PSMA-617. Ultimately, individualized dosimetry offers the potential to optimize therapeutic efficacy while minimizing toxicity and thereby improves long-term clinical outcomes. The use of ^161^Tb may also serve as a promising approach for other radionuclide therapies, including PRRT and FAP-targeted RLT [43, 44]. Further studies investigating efficacy, safety profile, and dosimetry of ^161^Tb-based radionuclide therapies, preferably in a prospective setting, are warranted.

Several limitations of the present study should be considered. The relatively small cohort size and the heterogeneity of the study population restrict the generalizability of the findings and warrant confirmation in larger, prospective studies. Furthermore, all patients had undergone multiple prior cycles of PSMA RLT, which may have affected tumor and organ kinetics as well as PSMA expression. Therefore, the dosimetric results should be interpreted within the context of a heavily RLT-pretreated population and cannot be assumed to be directly applicable to RLT-naïve patients. Due to the retrospective nature of the study and the missing of repeated blood samples, red bone marrow dosimetry was not feasible. With respect to the dosimetry methodology, the clinical protocol did not include more than three imaging time points due to increased patient burden associated with the time-intensive data acquisition required for dosimetry. In addition, lesion selection was constrained by predefined imaging and segmentation criteria, introducing the potential for selection bias in the lesion-level analyses.

## Conclusion

The administration of [^161^Tb]Tb-PSMA-617 delivers high absorbed radiation doses to tumor lesions while maintaining tolerable absorbed doses to critical organs at risk, thereby providing favorable tumor-to-normal-organ dose ratios. Dosimetric data confirms the potential of ^161^Tb as a promising radionuclide for PSMA-targeted radioligand therapy. Future prospective investigations in larger cohorts are needed to validate these findings.

## Supplementary Information

Below is the link to the electronic supplementary material.

Supplementary Material 1

## Data Availability

The datasets generated and analyzed during the current study are not publicly available but can be provided by the corresponding author on reasonable request.
