# Supplementary material for: Organ and tumor dosimetry of [161Tb]Tb-PSMA-617 radioligand therapy in patients with mCRPC
Source: Eur J Nucl Med Mol Imaging. 2026 Sep 8;53(13):8519–30. doi: 10.1007/s00259-026-08171-z (PMC13727920; doi:10.1007/s00259-026-08171-z)
Supplement: Supplementary file 1 [file 259_2026_8171_MOESM1_ESM.docx]

**Supplementary Material**

**Figure S1:** Radiation dose deposited during treatment cycles 1 and 2 of [^161^Tb]Tb-PSMA-617 RLT calculated for the entire patient cohort. **A**: to normal organs, **B:** to tumor lesions. Statistical analysis revealed only non-significant differences between the treatment cycles (kidneys: *p*>0.999, liver *p*=0.183, parotid glands *p*=0.250 and submandibular glands *p*=0.438, tumor lesions: *p*=0.102).

**Table S1:** Mean serum PSA determined before and after the initial cycle of [^161^Tb]Tb-PSMA-617 RLT for the three groups of patients classified according to PCWG3 criteria (PSA decrease of **≥** 50%: partial remission (PR); decrease < 50% or increase < 25%: stable disease (SD); increase **≥** 25%: progressive disease (PD)) and for the entire cohort of the study.

| ***Patient group according to*** | ***No. of patients*** | ***initial PSA*** | ***PSA response*** | ***%PSA change*** |
| --- | --- | --- | --- | --- |
| ***PCWG3 criteria*** |  |  |  |  |
| ***All*** | *15* | *259.2 ± 525.8* | *346.9 ± 910.0* | *-18.7 ± 52.5* |
| ***PR*** | *5* | *111.8 ± 182.0* | *47.4 ± 86.9* | *-71.6 ± 14.9* |
| ***SD*** | *6* | *120.8 ± 157.3* | *90.3 ± 129.7* | *-26.5 ± 15.0* |
| ***PD*** | *4* | *651.2 ± 1106* | *1106.3 ± 1511.0* | *+59.0 ± 10.1* |
